# Association of Gabapentin Use With Pain Control and Feeding Tube Placement Among Patients With Head and Neck Cancer Receiving Chemoradiotherapy

**DOI:** 10.1001/jamanetworkopen.2022.12900

**Published:** 2022-05-18

**Authors:** Sung Jun Ma, Katy Wang, Austin J. Iovoli, Kristopher Attwood, Gregory Hermann, Mark Farrugia, Anurag K. Singh

**Affiliations:** 1 Department of Radiation Medicine, Roswell Park Comprehensive Cancer Center, Buffalo, New York; 2 Department of Biostatistics and Bioinformatics, Roswell Park Comprehensive Cancer Center, Buffalo, New York; 3 Department of Radiation Oncology, OSF HealthCare Saint Francis Medical Center, University of Illinois College of Medicine at Peoria, Peoria, Illinois

## Abstract

This comparative-effectiveness study uses data from 2 clinical trials to evaluate whether the use of gabapentin for pain management is associated with less opioid use and feeding tube placement among adult patients with head and neck cancer receiving chemoradiotherapy.

## Introduction

Almost one-half of patients treated with radiotherapy (RT) for oropharyngeal cancer require chronic opioid use to manage oral mucositis (OM) pain.^1^ Given the paucity of level 1 evidence for OM pain management, current guidelines allow consideration of prophylactic gabapentin.^2,3^ However, the optimal dose remains unclear. In this comparative effectiveness study, we performed a secondary analysis of 2 clinical trials conducted by this research team, hypothesizing that a higher dose of gabapentin would be associated with less opioid use and feeding tube (FT) placement.

## Methods

Eligible patients in both studies received concurrent chemoradiotherapy for nonmetastatic squamous cell carcinoma of the head and neck and prophylactic oral gabapentin (titrated to 900 mg vs 2700 mg daily in 1 study^4^ and 3600 mg daily in the other study). Using the 3600 mg cohort as the reference group, we performed a multivariable competing risks analysis to evaluate time to first use of opioids and a logistic regression analysis to assess FT placement, adjusted for baseline characteristics. This study followed the International Society for Pharmacoeconomics and Outcomes Research (ISPOR) reporting guideline. Additional information is available in eMethods in the Supplement.

## Results

Among 92 patients, the median age was 62.1 years (IQR, 55.4-66.4 years); 82 patients were male (89.1%), and 10 (10.9%) were female. Baseline cohort characteristics were well balanced (Table). Most patients tolerated gabapentin per protocol. For example, adverse effects from gabapentin, such as dizziness and drowsiness, were only reported in 1 patient (3.1%) in the 3600 mg cohort (grade 3 dizziness) and 2 patients (6.3%) in the 3600 mg cohort (gabapentin was discontinued because of nausea and vomiting partly associated with RT and low adherence to oral hygiene).

**Table.** Baseline Participant Characteristics

Characteristic	No. (%)			*P* value
	Gabapentin, 3600 mg	Gabapentin, 2700 mg	Gabapentin, 900 mg	
Total participants, No.	32	31	29	NA
Age, median (IQR), y	63.0 (56.8-68.8)	62.0 (55.2-65.1)	61.9 (54.3-64.6)	.43
Sex				
Male	28 (87.5)	27 (87.1)	27 (93.1)	.77
Female	4 (12.5)	4 (12.9)	2 (6.9)	
Race				
Racial minority group^a^	1 (3.1)	1 (3.2)	0	>.99
White	31 (96.9)	30 (96.8)	29 (100)	
ECOG performance status				
0	23 (71.9)	23 (74.2)	23 (79.3)	.76
1	8 (25.0)	7 (22.6)	6 (20.7)	
2	1 (3.1)	1 (3.2)	0	
BMI, median (IQR)	29.3 (26.2-32.4)	28.6 (24.3-31.3)	29.9 (27.6-32.2)	.45
Primary disease site				
Oropharynx	27 (84.4)	22 (71.0)	21 (72.4)	.38
Other	5 (15.6)	9 (29.0)	8 (27.6)	
*AJCC-7* cancer stage				
II	0	2 (6.5)	0	.25
III	5 (15.6)	9 (29.0)	7 (24.1)	
IV	27 (84.4)	20 (64.5)	22 (75.9)	
Feeding tube before radiotherapy				
No	31 (96.9)	29 (93.5)	29 (100)	.65
Yes	1 (3.1)	2 (6.5)	0	
Neck radiotherapy				
Unilateral	10 (31.3)	4 (12.9)	5 (17.2)	.19
Bilateral	22 (68.8)	27 (87.1)	24 (82.8)	

Abbreviations: *AJCC-7*, *AJCC Cancer Staging Manual* (7th edition); BMI, body mass index (calculated as weight in kilograms divided by height in meters squared); ECOG, Eastern Cooperative Oncology Group; NA, not applicable.

^a^ Racial minority groups included Asian or Pacific Islander, Black or African American, and unreported (participants declined to report race).

The multivariable competing risks model revealed the time to first opioid use for additional pain control was greatest in the 3600 mg cohort (Figure). The proportion of patients requiring opioids during RT was also smallest in the 3600 mg cohort (12 patients [37.5%]) vs the 900 mg cohort (27 patients [93.1%]) and the 2700 mg cohort (19 patients [61.3%]; *P* < .001). Multivariable logistic regression analysis revealed that, compared with the 3600 mg cohort (3 patients [9.4%]), the 2700 mg cohort (14 patients [45.2%]) had significantly greater odds of FT placement during RT (adjusted odds ratio, 9.9; 95% CI, 2.1-75.7; *P* = .009); however, the odds were not significantly greater in the 900 mg cohort (6 patients [20.7%]; adjusted odds ratio, 3.6; 95% CI, 0.7-28.1; *P* = .15).

**Figure.**
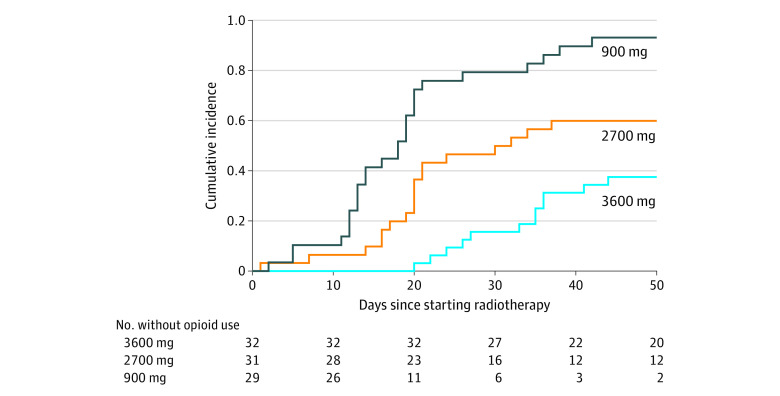
Cumulative Incidence of First Use of Opioid Medication During Radiotherapy Over Time Multivariable competing risks analysis among cohorts receiving daily doses of gabapentin, 900 mg, 2700 mg, or 3600 mg (reference cohort). Adjusted for age, sex, performance status, body mass index, pretreatment feeding tube placement, primary disease site, cancer stage, and receipt of unilateral vs bilateral neck radiotherapy. The adjusted hazard ratios were 6.6 (95% CI, 3.2-13.6; *P* < .001) for the 900 mg cohort and 2.4 (95% CI, 1.2-4.8; *P* = .01) for the 2700 mg cohort compared with the 3600 mg cohort.

## Discussion

This study is, to our knowledge, the first evaluation of prospective data to suggest that higher doses of gabapentin (900-3600 mg daily) are well tolerated and associated with delayed time to first opioid use for OM pain control. Consistent with our study, Smith et al^5^ found that gabapentin decreased pain during RT, but the dose was not increased beyond 900 mg for most patients because early pain relief occurred at this dose; most patients in our study tolerated 3600 mg. Cook et al^6^ found no benefit to using 1800 mg of gabapentin daily; patients receiving gabapentin had a higher rate of FT placement than patients receiving placebo (62.1% vs 20.7%). In our study, FT placement rates were comparable between subgroups of the 3600 mg and 900 mg cohorts in which methadone was used for rescue treatment, whereas the rate was significantly worse in the subgroup of the 2700 mg cohort in which hydrocodone and fentanyl were used for rescue treatment. Methadone has been reported to be more effective for OM pain control than hydrocodone and fentanyl.^4^ These findings suggest the need for FT placement may not be associated with gabapentin dose alone but with the overall efficacy of pain management, including rescue regimens.

Despite having well-balanced baseline cohort characteristics, this study remains an unplanned secondary analysis of 2 consecutive prospective clinical trials. Nevertheless, prophylactic gabapentin with 3600 mg daily was well tolerated and eliminated the need for opioids during RT in 62.5% of patients. Although gabapentin, 3600 mg, daily has been adopted as the standard regimen of the Roswell Park Comprehensive Cancer Center, additional studies are warranted to further investigate its role in pain control.

## Notes

Supplement. **eMethods.** Study Approval, Participants, Treatment, Data Collection, and Statistical Analysis
