# Supplementary material for: Association of Gabapentin Use With Pain Control and Feeding Tube Placement Among Patients With Head and Neck Cancer Receiving Chemoradiotherapy
Source: JAMA Netw Open. 2022 May 18;5(5):e2212900. doi: 10.1001/jamanetworkopen.2022.12900 (PMC9118041; doi:10.1001/jamanetworkopen.2022.12900)
Supplement: Supplement. — eMethods. Study Approval, Participants, Treatment, Data Collection, and Statistical Analysis [file jamanetwopen-e2212900-s001.pdf]

Supplementary Online Content

Ma SJ, Wang K, Iovoli AJ, et al. Association of gabapentin use with pain control and feeding tube placement among patients with head and neck cancer receiving chemoradiotherapy. *JAMA Netw Open*. 2022;5(5):e2212900. doi:10.1001/jamanetworkopen.2022.12900

eMethods. Study Approval, Participants, Treatment, Data Collection, and Statistical Analysis

This supplementary material has been provided by the authors to give readers additional information about their work.

© 2022 Ma SJ et al. *JAMA Network Open*.

eMethods. Study Approval, Participants, Treatment, Data Collection, and Statistical Analysis

Both single-institution, consecutive, prospective randomized trials (ClinicalTrials.gov identifier NCT02531906 and NCT03574792) were approved by the institutional review board (IRB) at the Roswell Park Comprehensive Cancer Center (I-262314 and I-61117). Written informed consent was obtained for all participants. Our study follows the International Society for Pharmacoeconomics and Outcomes Research (ISPOR) reporting guideline.

Patients were accrued from April 2015 to March 2021. Data analysis was performed from September 2021 to December 2021. Follow up was until 6 months after completing radiation therapy, with optional follow up at 9 and 12 months. Eligible patients for both studies were (1) 18 years or older, (2) diagnosed with pathologically proven, stage II-IV (American Joint Committee on Cancer 7th edition) squamous cell carcinoma of the head and neck undergoing definitive-intent chemoradiation, (3) had adequate renal function to receive platinum-based chemotherapy, (4) had an Eastern Cooperative Oncology Group performance status 2 or less, (5) and had the ability to swallow or tolerate medications through feeding tube. They were excluded if they (1) had prior treatments for head and neck cancer, (2) had recurrent or metastatic head and neck malignancies, (3) had pre-existing chronic pain or neuropathy, (4) were currently under substance abuse programs, (5) were on dialysis or had transplanted organs, and (6) had uncontrolled medical comorbidities. Additional exclusion criteria for I-61117 included those who (1) were taking any medication that may increase a risk of serotonin syndrome and (2) had acute narrow-angle glaucoma.

All patients completed a staging workup with computed tomography (CT) of the head and neck with contrast and/or positron emission tomography-computed tomography (PET/CT). All patients underwent definitive radiation therapy with an intensity modulated radiation therapy (IMRT; 70 Gy/35 fractions to the primary tumor, 56 Gy/35 fractions to elective lymph nodes) with concurrent cisplatin-based chemotherapy (weekly or every 3 weeks).

Patients were randomized in 1:1 ratio in permuted blocks of 6 patients each, which was masked to the investigators, and they were stratified by unilateral versus bilateral neck radiation. Due to the nature of the intervention using gabapentin, patients and investigators were not blinded.

All patients received oral gabapentin, starting at 300 mg daily on day 1 and gradually escalating by adding 300 mg to the total daily dose (eg 300 mg twice a day on day 2, 300 mg three times a day on day 3). Gabapentin was titrated up to 300 mg three times a day for the 900 mg cohort over the course of a minimum of 3 days, 900 mg three times a day for the 2700 mg cohort over the course of a minimum of 9 days, and 1200 mg three times a day for the 3600 mg cohort over the course of a minimum of 12 days. The 900 mg and 3600 mg cohorts used methadone 2-15 mg three times a day as the first line rescue regimen (followed by oxycodone 5-10 mg three times a day as the second line regimen) as needed for breakthrough pain. The 2700 mg cohort used hydrocodone 7.5 mg and acetaminophen 325 mg up to four times a day (followed by fentanyl transdermal patch titrated up to 100 μg/hour as the second line regimen). For patients with impaired renal function during radiation therapy, their gabapentin doses were adjusted accordingly. After shared decision making between patients and clinicians, gabapentin was tapered off among patients who reported to be unable to tolerate it due to significant side effects or symptom burden.

All patients received educational materials and were encouraged on oral hygiene, hydration, and nutrition. Patients were encouraged to gargle with a saline/baking soda mouthwash rinse as

© 2022 Ma SJ et al. *JAMA Network Open*.

many times as possible per day (eg 20 times) and use a compounded elixir of diphenhydramine, xylocaine, and antacid in a 1:1:1 ratio 4 times per day for pain.

All study data were collected and managed using Research Electronic Data Capture (REDCap), hosted at the Roswell Park Comprehensive Cancer Center. Baseline patient demographics and tumor characteristics were collected. Patients were evaluated for pain level, the extent of oral mucositis, weight changes, and feeding tube usage at baseline, weekly during radiation therapy and at the completion of radiation therapy. Feeding tube placement was performed with multi-disciplinary evaluation of factors including patients' nutritional and functional status, speech and swallow evaluation, and shared discussions among patients, family members, caregivers, and physicians.

Primary endpoint of this secondary analysis was time to first opioid and feeding tube placement. Kruskal-Wallis and Fisher exact tests were used to compare baseline characteristics and the proportion of patients requiring opioids during radiation therapy. With the 3600 mg cohort as a reference, multivariable competing risk and logistic regressions were performed to evaluate time to first opioids and feeding tube placement, respectively. Multivariable models were adjusted for baseline characteristics, including age, gender, performance status, body mass index, pre-treatment feeding tube placement, primary disease site, staging, and unilateral versus bilateral neck radiation.

Bonferroni correction was used for multiple comparisons (the 3600 mg cohort vs the 2700 mg cohort; the 3600 mg cohort vs the 900 mg cohort). All statistical tests were two-sided, and p values lower than 0.025 were considered statistically significant. All analyses were performed using R (version 4.0.3, R Project for Statistical Computing, Vienna, Austria).

© 2022 Ma SJ et al. *JAMA Network Open*.
